# Comparative Evaluation of the Efficacy of Combined Intramedullary Pinning with K-Wires Pinning in the Treatment of Fifth Metacarpal Neck Fractures versus Conventional Techniques—K-Wires Pinning and Intramedullary Pinning

**DOI:** 10.3390/medicina59111944

**Published:** 2023-11-03

**Authors:** Dong-Eun Kim, Tong-Joo Lee, Yeop Na, Ye-Geon Noh

**Affiliations:** Department of Orthopaedic Surgery, Inha University Hospital, Incheon 22332, Republic of Korea; apenfkrl@gmail.com (D.-E.K.); 971111r@naver.com (Y.-G.N.)

**Keywords:** hand, fifth metacarpal neck, K-wire pinning, intramedullary pinning

## Abstract

*Background and Objectives*: Since the neck is the weakest part of the metacarpals, the most common metacarpal fracture is a neck fracture, a type which accounts for 38% of all hand fractures. Such fractures can be fixed using a variety of conventional techniques, including intramedullary pinning and K-wire pinning. However, conventional techniques involve complications, such as angulation, stiffness, and rotational deformity. The purpose of this study was to compare the usefulness of our new technique, combined intramedullary pinning with K-wire pinning (IPKP), with those of intramedullary pinning (IP) and K-wire pinning (KP). *Materials and Methods*: This was a single-center, randomized controlled trial conducted between January 2005 and April 2023. A total of 158 patients with acute displaced fractures of the fifth-metacarpal neck were randomly assigned to either the IPKP group (*n* = 48), the KP group (*n* = 60), or the IP group (*n* = 50). We radiographically evaluated angulation and shortening in three visits: pre-operatively, post-operatively, and at a 1-year follow-up. We clinically evaluated the ranges of motion and Quick-DASH scores to assess daily living performance and the cosmetic scores, using the SBSES score, to assess patients’ satisfaction with their cosmetic outcomes. *Results:* The IPKP group was superior to the KP group and the IP group regarding radiographical and clinical assessments at the 1-year follow-up visit. The angulation was 15.7° (±7.7) in the KP group, 17.0° (±5.9) in the IP group, and 12.6° (±2.5) in the IPKP group (*p* < 0.001) at the 1-year follow-up visit. The shortening was 0.9 mm (±0.3) in the KP group, 1.4 mm (±0.2) in the IP group, and 0.4 mm (±0.1) in the IPKP group (m < 0.001) at the 1-year follow-up visit. The TAM was 272.6° (±17.5) in the KP group, 271.1° (±18.0) in the IP group, and 274.1° (±14.9) in the IPKP group (*p* = 0.42). Four patients (6.6%) in the KP group and two patients (4%) in the IP group were reported as having stiffness, while no patients were found to have stiffness in the IPKP group. The average Quick-DASH score was 2.3 (±0.5) in the KP group, 2.5 (±0.4) in the IP group, and 1.9 (±0.4) in the IPKP group (*p* > 0.05). The average cosmetic score was 3.7 (±1.2) in the KP group, 3.8 (±0.9) in the IP group, and 4.7 (±0.8) in the IPKP group (*p* < 0.001). A complication involving nonunion occurred in one case (1.6%) in the KP group, while there were three cases (6%) of rotational deformity in the IP groups. *Conclusions*: With the IPKP technique, accurate reduction can be achieved to improve hand function and cosmetic outcomes.

## 1. Introduction

Boxer’s fractures, referring to fractures of the fifth-metacarpal neck, represent a commonly encountered injury in the domain of orthopedic practice. These fractures frequently arise because of a forceful impact upon a clenched fist, resulting in distinct angulation and displacement of the affected bone [1]. The management of these fractures seeks to facilitate the restoration of hand function, reduce pain, and ensure an early return to activities [2]. Traditional treatment methods include K-wire pinning and intramedullary pinning, with each demonstrating certain drawbacks, such as angulation, reduction loss, metacarpal neck shortening, and reduced range of motion (ROM) [3]. Thus, in this paper, we propose a new surgical technique, termed intramedullary pinning combined with K-wire pinning (IPKP), which holds the potential to provide augmented stability and superior clinical outcomes.

Presently, K-wire pinning (KP) and intramedullary pinning (IP) are among the most commonly employed surgical techniques for metacarpal neck fractures, although they have been associated with diverse complications. KP may induce angulations or reduction loss, which can reduce hand function during daily activities [2]. Similarly, IP may lead to issues such as shortening, rotational deformity, and reduced range of motion (ROM). The consequences of metacarpal neck shortening can manifest as extensor lag in the fifth finger [4], while rotational deformities can impede patients from forming a firm fist, consequently impacting their daily lives and influencing their Quick-DASH scores and cosmetic scores [5]. In this paper, we present a new surgical technique aimed at solving these complications and enhancing both hand function and cosmetic aspects in patients with metacarpal neck fractures. Among the numerous emerging techniques for minimizing complications, we explored new fixation techniques, including a non-invasive approach intended to minimize soft-tissue injury. Consequently, we propose a technique that builds upon the conventional methods by combining intramedullary pinning with K-wire pinning to reduce the incidence of such complications.

## 2. Materials and Methods

### 2.1. Study Design

A comparative evaluation study was undertaken to assess the relative efficacy of three distinct treatment modalities for fractures in the fifth-metacarpal neck, namely, the K-wire pinning (KP) method, the intramedullary pinning (IP) method, and the combined intramedullary pinning with K-wire pinning (IPKP) method. Surgery using one of these three methods was decided upon based on the surgeon’s preference. Conducted as a retrospective study at a single center, this study’s duration spanned from January 2005 to April 2023, utilizing medical records and radiographic images as the primary data sources. The inclusion criteria for this study sought patients aged 18 years or older with a diagnosis of a fifth-metacarpal neck fracture exhibiting angulations exceeding 40° (the normal range of angulation is <15°) [5,6]. The availability of complete medical records and radiographic images after a minimum follow-up of 1 year was also considered.

Patients who met the inclusion criteria were included in this study, regardless of gender, underlying medical conditions, or previous hand injuries. However, patients meeting any of the following criteria were excluded: any injuries to tendons, ligaments, vessels, or nerves of the ipsilateral upper limbs; multiple fragmentary fractures; combined operations; open fractures; and non-cooperative patients.

In our series, 90 of the 158 patients were male. The average age was 45.9 ± 8.3 (18 to 69). The right hand was affected in 94 cases and the left hand in 64. Statistically, there were no significant differences in the demographics of the three groups (*p* > 0.05).

### 2.2. Methods

All patients were clinically assessed using the Quick-DASH questionnaire, and a cosmetic score was obtained using the SBSES score at 12 months post-operation. The active ranges of motion (ROM) of the MP, PIP, and DIP joints were recorded using a flat, 5.5-inch-long, stainless-steel finger goniometer (Baseline^®^, Fabrication Enterprise Inc., White Plains, NY, USA), from which the total active motion (TAM) was calculated. Following the guidelines of the American Association of Orthopedic Surgery, full range of motion (ROM) was measured at 280 degrees. For this study, we chose to classify patients experiencing a ROM of 70% or less as exhibiting stiffness. Radiographic images were assessed by two orthopedic surgeons and acquired preoperatively, immediately postoperatively, and 12 months after surgery. The images included an anteroposterior (AP) view, a lateral oblique hand X-ray, and the dorsal apex angle (DAA) to determine angulation and shortening. Rotational deformity was clinically assessed with overlapping fingers, while a clenched fist showed a considerable degree of rotation. Furthermore, alignment could be assessed by examining the hand with the MCP and PIP joints in flexion and DIP joints extended. If lines drawn along the digits did not converge toward the others, we considered it a rotational deformity [7]. To calculate the shortening of the 5th metacarpal, a line was drawn through the most distal point of the heads of the neighboring 3rd and 4th metacarpals. The shortening was defined as the distance from this line to the most distal point of the fractured 5th metacarpal bone. The patients were instructed to complete the questionnaires at our clinic at 1-year follow-up visits. Functional outcomes and patient-reported disabilities were assessed using the Quick Disabilities of the Arm, Shoulder, and Hand (Quick-DASH) score. Lower scores on these assessments indicate superior functional outcomes and reduced disability. Additionally, patients rated their cosmetic satisfaction on a scale from 1 to 5, where higher scores denoted greater contentment with their cosmetic outlooks.

### 2.3. Surgical Technique

**Figure 1:**
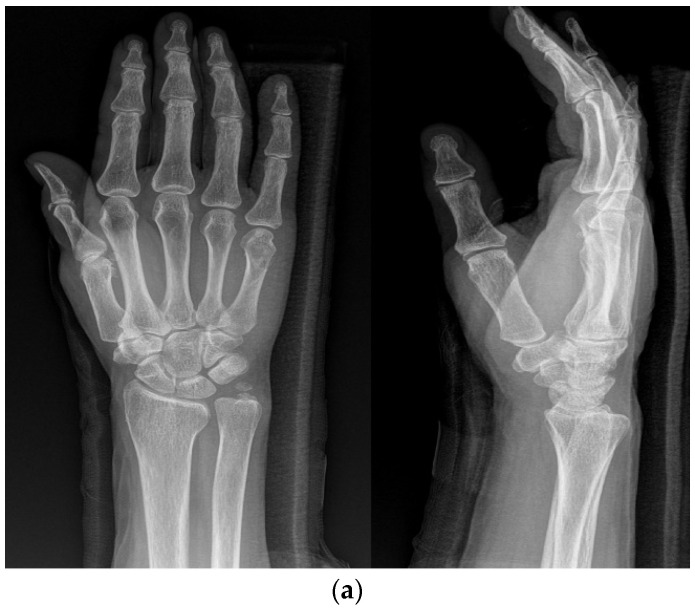

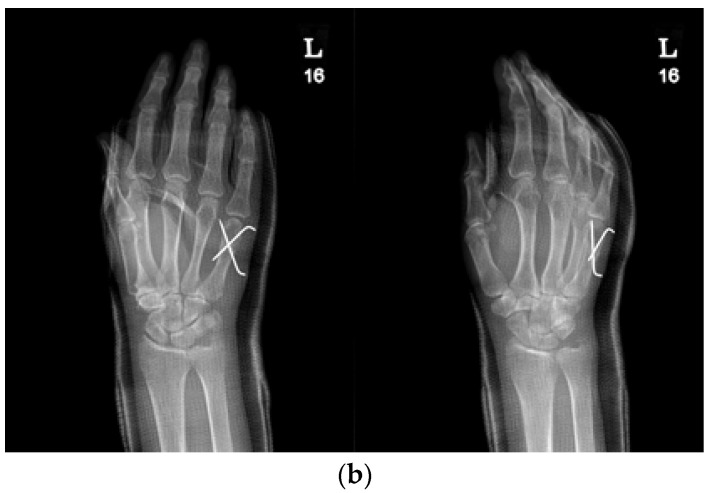
(**a**) Radiograph of a 70-year-old female patient with displaced little-finger metacarpal neck fracture of the left hand. (**b**) Radiograph of a 70-year-old female patient who underwent the KP method using two K-wires.

The KP method involved the insertion of two K-wires across the fracture site to stabilize the fracture. The number and size of K-wires used were determined based on the surgeon’s preference and the specific characteristics of the fracture (Figure 1a,b).

**Figure 2:**
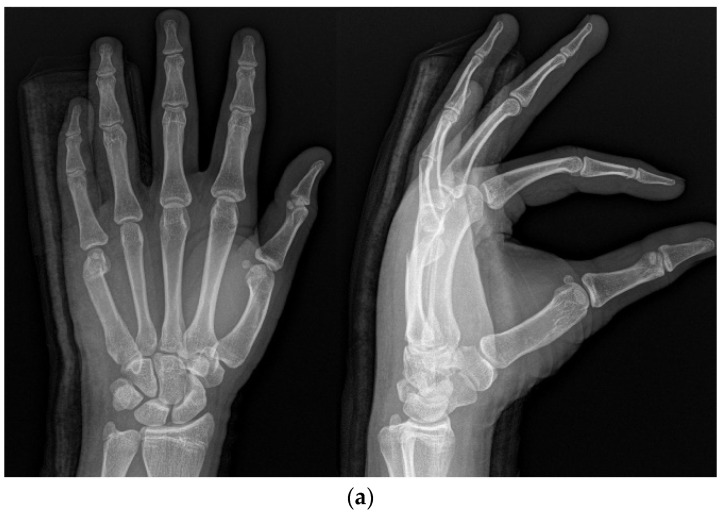

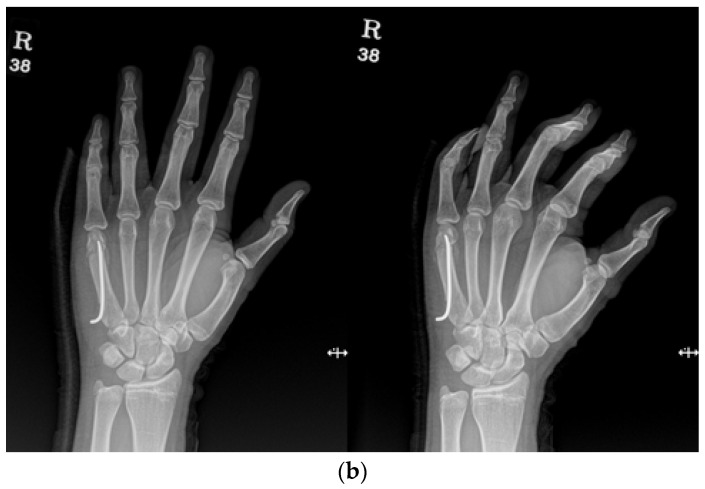
(**a**) Radiograph of a 16-year-old male patient with displaced little-finger metacarpal neck fracture. (**b**) Radiograph of a 16-year-old male patient who underwent the IP method using one intramedullary nail in the anterograde direction.

The IP method involved the insertion of an intramedullary nail in the anterograde direction. The entry point was selected at the base of the ulnar dorsal border of the metacarpal using a needle under imaging guidance. A small hole was made with a drill, into which the operator inserted the nail. The fracture was reduced via maneuver, and the nail passed across the fracture part (Figure 2a,b).

**Figure 3:**
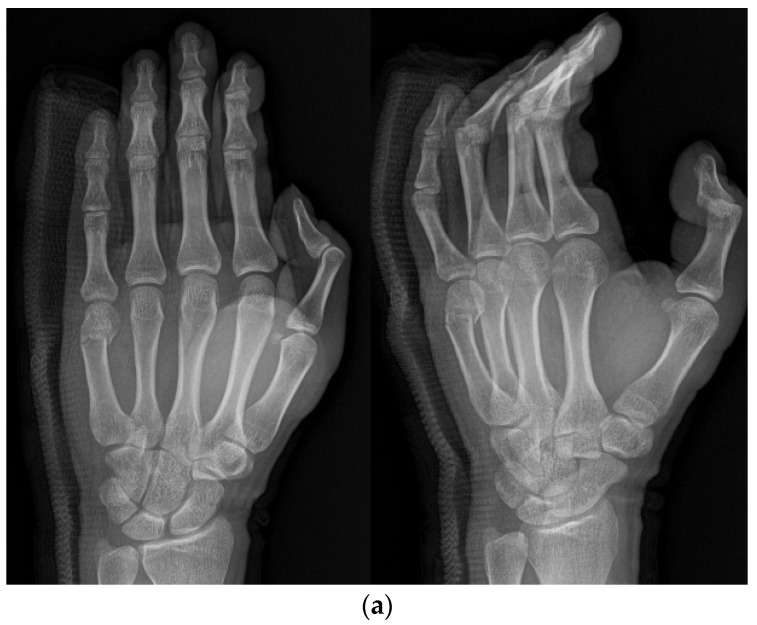

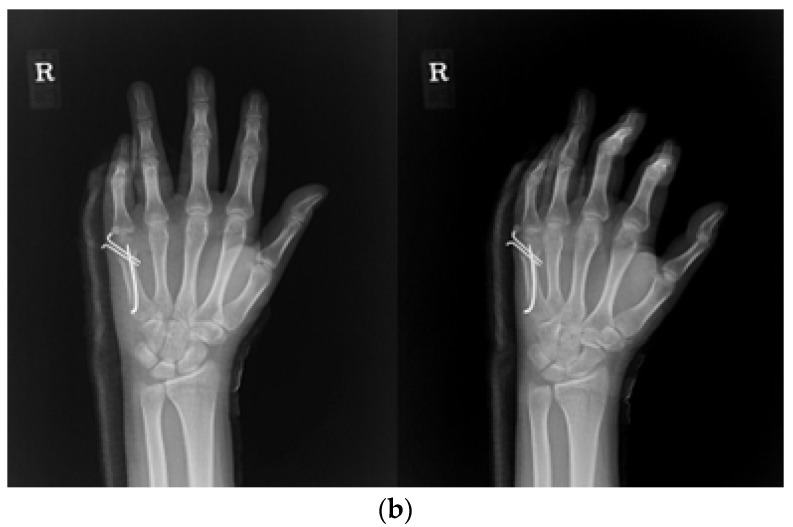
(**a**) Radiograph of an 18-year-old male patient with displaced little-finger metacarpal neck fracture. (**b**) Radiograph of a 16-year-old male patient who underwent the IPKP method using one intramedullary nail and two additional K-wires.

The combined method involved the placement of an intramedullary pin followed by parallel K-wire fixation. Initially, an intramedullary nail was inserted into the metacarpal bone to stabilize the fracture, typically through the base. Following intramedullary pinning, K-wires were placed across the distal part of the intramedullary nail, and the pin was inserted halfway through the dorsal aspect of the nail, touching the nail and making an additional accurate reduction possible. Another pin was inserted on the volar aspect of the nail (Figure 3a,b).

All three methods involved postoperative immobilization using a splint for 4 weeks. We encouraged patients to practice ROM after taking off splints. The pins were removed after 6 weeks, postoperatively.

### 2.4. Statistical Analysis

We prospectively evaluated patients using a 2-sided significance level of 0.05 and a power of 80%. The sample size was calculated according to the outcome of angulation. A power analysis revealed that to detect an angulation of 12.6 degrees, with a standard deviation of 2.5 degrees and a type-I error rate of 0.05, a power of 0.8 and a minimum of 60 patients were needed per treatment group. An ANOVA was performed to determine the results of clinical and radiological outcomes for analysis. Statistical significance was set at *p* < 0.05, and each *p*-value was corrected via a Bonferroni correction test. All statistical analyses were performed using SPSS version 25 (IBM Corporation, Armonk, NY, USA).

## 3. Results

A total of 158 patients with metacarpal neck fractures were included in this study, with 60 patients treated using the KP method, 50 patients treated using the IP method, and 48 patients treated using the IPKP method. The demographic characteristics of the three groups exhibited similarities in age and gender distribution. There were no differences in radiological features in the pre-operative X-rays between the three groups, as shown in Table 1. On average, the mean duration of surgery for the IPKP method was 3 to 5 min longer than those for the other methods; however, these differences were not statistically significant (*p* = 0.07).

The assessment of angulation degrees was conducted based on radiographic images acquired pre-operatively, post-operatively, and at 1-year follow-up visits. The angulation measurements immediately after surgery were as follows: 13.1° (±5.6) in the KP group, 13.1° (±5.1) in the IP group, and 10.3° (±4.0) in the IPKP group (*p* = 0.04), However, at the 1-year follow-up mark, patients in the KP group exhibited an average angulation of 15.7° (±7.7), while in the IP group, the average angulation measured 17.0° (±5.9). The IPKP group displayed a substantially lower average angulation, of 12.6° (±2.5). The observed difference in the angulation degrees between the three groups was statistically significant (*p* < 0.05), indicating the superiority of our new technique.

The assessment of shortening was conducted based on radiographic images obtained immediately postoperatively and at 1-year follow-up visits. Upon immediate postoperative evaluation, the observed shortening measurements were as follows: 0.5 mm (±0.5) in the KP group, 0.7 mm (±0.4) in the IP group, and 0.3 mm (±0.2) in the IPKP group (*p* < 0.001). However, at the 1-year follow-up visits, patients in the KP group displayed an average shortening of 0.9 (±0.3) mm, while in the IP group, the average shortening measured 1.4 mm (±0.2). The IPKP group exhibited a considerably lower average shortening, of 0.4 (±0.1) mm. The observed variance in shortening degrees between the three groups was deemed statistically significant at the 1-year follow-up visits (*p* < 0.05), indicating the superiority of the IPKP method in minimizing shortening, compared with the other approaches.

At the 1-year follow-up visit, the range of motion (ROM) of the affected finger(s) was measured in degrees using goniometry. For comparative purposes, the flexion angles of the MCP joint, PIP joint, and DIP joint were added for comparison. The results show that the groups exhibited total active motion (TAM) measurements of 272.6° (±17.5) in the KP group, 271.1° (±18.0) in the IP group, and 274.1° (±14.9) in the IPKP group. While the inter-group comparison did not yield statistically significant differences (*p* > 0.05), an incidence of stiffness was reported for the following number of cases for each group: four patients (6.6%) in the KP group, two patients (4%) in the IP group, and none in the IPKP group. It is noteworthy that the IPKP group demonstrated nearly full ROM in comparison with the other groups, and no cases of stiffness were reported.

The mean Quick-DASH score was 2.3 (±0.5) in the KP group, 2.5 (±0.4) in the IP group, and 1.9 (±0.4) in the IPKP group. The between-group comparisons showed a statistically significant difference (*p* < 0.05).

The cosmetic score was 3.8 (±0.7) in the KP group, 3.8 (±0.7) in the IP group, and 4.7 (±0.5) in the IPKP group. The between-group comparisons showed a statistically significant difference (*p* < 0.05).

Several cases with complications were reported in the KP and IP groups. In particular, rotational deformity was observed in only three patients (6%) in the IP group, while no instances of rotational deformity were reported in the other groups. Furthermore, nonunion was reported by one patient (1.6%) in the KP group, necessitating revision surgery after a few months.

## 4. Discussion

Our IPKP method demonstrated superior outcomes compared with the conventional methods. At the 1-year follow-up visits, the average shortening measured 0.4 mm (±0.1), and the average angulation was 12.6° (±2.5), both exhibiting a statistically significant difference (*p* < 0.05). These findings support the notion that our new technique surpasses both the KP and IP methods in terms of efficacy. Moreover, the fact that the IPKP method’s immediate postoperative angulation and shortening was significantly lower than that of the IP or KP method, further reducing the distal fragment by using the joystick technique for more accurate reduction, shows the potential of our new technique. Additionally, the average Quick-DASH score for our new technique was 1.9 (±1.6), with the average total active motion (TAM) measuring 274.1° (±14.9). Although the range of motion (ROM) did not show a statistically significant difference (*p* > 0.05) when comparing all three groups (Table 1), a statistically significant difference (*p* < 0.05) was observed when we compared the KP group or IP group with the IPKP group separately, further affirming our new technique’s superiority.

Among the various conventional methods, internal fixation using plates is known to provide substantial mechanical strength; however, many complications have been documented. These complications encompass metacarpal head avascular necrosis, nonunion, severe tendon irritation, and a high incidence of stiffness attributable to excessive soft tissue damage, which, in turn, may lead to infections [8,9,10]. Open reduction and internal fixation can also result in adhesions in the joints and extensor tendons [2]. In contrast with plating methods, our new technique adopts a non-invasive approach to approach the fracture site, minimizing soft-tissue injury while ensuring accurate reduction.

Conventional K-wire pinning is associated with complications such as angulations and reduction loss [11,12]. A study conducted by Lee et al. [12] reported the recurrence of angulation, from 20.2° at a 6-month follow-up visit to 24.2° at the last follow-up visit, in cases using K-wire pinning for fixation. In contrast, our new technique displayed significantly improved radiological outcomes, with angulation measurements of 10.3° (±4.0) at immediate postoperative evaluation and 12.6° (±2.5) at the 1-year follow-up visits. In a study conducted by Choi et al. [13], transverse pinning using K-wires was employed in 39 patients, resulting in three cases (7.6%) of under-reduction and one case (2.5%) of nonunion. In contrast, our new technique exhibited no instances of these complications. These findings show the superior performance of the IPKP method, demonstrating lower complication rates and improved radiological outcomes compared with the KP method.

The conventional intramedullary pinning (IP) method is known to result in complications such as shortening and reduced range of motion (ROM) [2,10,13]. In a study by Langqing et al. [10], 32 patients underwent anterograde intramedullary pinning. It reported an average shortening of 2.12 mm at the 1-year follow-up visits. In comparison, our new technique demonstrated a lower immediate postoperative shortening, of 0.3 mm (±0.2), and at the 1-year follow-up visits, the average shortening measured 0.4 (±0.1) mm. These results underscore the advantage of our new technique in preserving the metacarpal neck length, compared with the conventional IP method.

Sherif et al. [2] conducted a study involving 80 patients who underwent intramedullary ante-grade pinning. Patients demonstrating a total active motion (TAM) higher than 221° were classified as having an excellent outcome, while those with a TAM between 121° and 221° were considered to have a good outcome. The study reported that 80% of patients treated with the IP method achieved an excellent outcome, and 10% achieved a good outcome. However, one patient (2.5%) experienced a wire backing out after they lifted a heavy object. In contrast, our new IPKP technique achieved an excellent outcome in an impressive 89% of patients, with no instances of wire pull-out complications. Furthermore, Riazuddin et al. [14] considered ante-grade intramedullary pinning in twenty patients and reported that two out of eighteen cases (11.1%) of fifth-metacarpal fractures exhibited a 15° extensor lag of the MCP and IP joint, indicating the limitation of the IP method in potentially causing limited ROM during the 1-year follow-up period. However, our study demonstrated an average of 85° flexion in the MCP joint, proving that our new technique can induce almost full ROM within the 1-year follow-up period. These results show the efficacy of our new technique in improving the range of motion, which consequently positively impacts hand performance in daily activities [15,16,17,18].

Recently, new techniques that bear a resemblance to conventional methods have emerged. Eisenschenk et al. [11] reported the use of two K-wires as an intramedullary pinning method in thirty patients, revealing two cases (6.6%) of incomplete fist closure and one case (3.3%) of a rotational error exceeding 5°. Additionally, three cases (10%) experienced shortening of greater than 1.5 mm. In contrast, our study showed no instances of rotational error with our new technique, and the average shortening measured 0.4 (±0.1) mm at the 1-year follow-up visits, with no patients exhibiting shortening exceeding 1.5 mm. Eisenschenk et al. [11] also reported that using a less technically demanding single-wire technique produces non-inferior clinical and radiological outcomes compared with the dual-wire approach. This proves that using more K-wires for intramedullary fixation is not the key to reaching accurate reduction. Instead, we achieved accurate reduction according to radiological findings using additional K-wire fixation to directly approach the distal fragment from the lateral aspect of the fifth-metacarpal neck, using the joystick technique. This approach seems to be the key to better clinical and radiological outcomes [19,20].

A. Zemirline et al. [20] suggested a K-wires technique involving intermetacarpal percutaneous pinning externally connected to the HK2 connector. However, the average angulation in 18 patients undergoing this surgery was 23.1°, whereas our new technique exhibited an average angulation of 12.6° (±2.5). Furthermore, the Quick-DASH score in their study averaged 13.7, while our new method displayed a superior average score of 1.9. Zemirline et al. [20] also reported one crucial complication, where a patient underwent osteomyelitis of the fifth and fourth metacarpals, which was later resolved with antibiotics. Recent new techniques continue to encounter complications such as shortening, rotational deformities, and infection. In contrast, our new technique surpasses the KP and IP methods in terms of mitigating such complications, thus presenting a promising option for fifth-metacarpal neck surgery.

The mean duration of surgery for our new technique was 5 min longer compared with using the IP method alone. This means our new technique required an average of 10.6 min for the IP method and an additional 5 min to insert the parallel pinning. In comparison, the KP method took 13.2 min on average, 3 min longer than our new technique. The insertion of additional parallel pinning proved beneficial in achieving a more accurate reduction, leading to improved clinical outcomes [21,22,23,24,25]. Choi et al. [26] emphasized that, due to the angulation of the fracture site, fragments can cause cosmetic deformity of the metacarpal head collision even after reduction is performed. Consequently, patients are increasingly demanding better cosmetic outcomes after surgery [27,28]. We firmly believe that achieving a more accurate reduction can effectively prevent cosmetic deformities. By performing a noninvasive approach on the surgical sites, we successfully resolved cosmetic deformities, as evidenced by the significant improvements in patients’ cosmetic scores (*p* < 0.05). While the mean duration of surgery did not exhibit a significant difference (*p* > 0.05) between the three methods, our approach holds great potential for treating fifth-metacarpal neck fractures, given its capacity to achieve accurate reduction and address cosmetic concerns.

## 5. Limitation

First, since we did not examine computed tomography (CT) scans in our patients, we had to clinically evaluate rotational deformity instead of comparing radiological images. Comparing immediate postoperative and 1-year follow-up CT scans would have led to a more accurate comparison, which might have provided a more precise understanding of our new technique. Second, this was a retrospective study. Therefore, it was difficult to perform direct comparisons with other studies. Third, the number of patients who underwent our new technique does not match those of conventional groups. We developed this technique recently, which provided us with fewer surgery cases with which to make precise comparisons. Another limitation of this study is that we did not consider bouquet pinning. Moreover, inserting K-wires using an anterograde approach might have led to ideal outcomes. Comparing our new technique with this method might have generated more meaningful statistics and outcomes. Despite several limitations, our study is the first to report a new method that combines intramedullary pinning with K-wire pinning. It will aid in the selection of surgical techniques for fifth-metacarpal neck fractures.

## 6. Conclusions

In our experience, our new technique is a stable, non-invasive fixation method that provides fewer complications than do conventional methods in fifth-metacarpal neck fractures. All patients in the study group had satisfactory clinical and radiographic outcomes. Our results provide a new technique option for hand surgeons when choosing treatment for fifth-metacarpal neck fractures.

## Figures and Tables

**Table 1:** Clinical and radiological result data for three groups: KP, IP, and IPKP. KP—K-wire pinning method; IP—intramedullary pinning method; IPKP—combined intramedullary pinning with K-wire pinning method.

Variable	KP (n = 60)	IP (n = 50)	IPKP (n = 48)	*p*-Value
Quick-DASH	2.3 (±2.1)	2.5 (±1.7)	1.9 (±1.6)	<0.001
ROM (TAM)	272.6° (±17.5)	271.1° (±18.0)	274.1° (±14.9)	0.42
Angulation (Pre-op)	42.5° (±3.7)	40.8° (±4.4)	43.6° (±5.3)	0.09
Angulation (Immediate)	13.1° (±5.6)	13.1° (±5.1)	10.3° (±4.0)	0.04
Angulation (1 year f/u)	15.7° (±7.7)	17.0° (±5.9)	12.6° (±2.5)	<0.001
Shortening (Pre-op)	2.8 mm (±0.3)	2.8 mm (±0.4)	2.6 mm (±0.4)	0.26
Shortening (Immediate)	0.5 mm (±0.5)	0.7 mm (±0.4)	0.3 mm (±0.2)	<0.001
Shortening (1 year f/u)	0.9 mm (±0.3)	1.4 mm (±0.2)	0.4 mm (±0.1)	<0.001
Duration of surgery (min)	13.2 (±3.2)	10.6 (±2.5)	15.3 (±3.1)	0.07
Cosmetic score	3.7 (±1.2)	3.8 (±0.9)	4.7 (±0.8)	<0.001

The values are given as means. TAM—total active motion.

## Data Availability

The data used and analyzed during this study are available from the corresponding author upon reasonable request.
